# Case of Removal of the Human Spleen, without Injury or Derangement of the Animal Economy

**Published:** 1816-01

**Authors:** E. O'Brien

**Affiliations:** Surgeon, Royal Navy


### *Case of Removal of the Human Spleen, without Injury or Derangement of the Animal Economy.*

By E. O'Brien, Esq. Surgeon, Royal Navy.

( Communicated by Mr. Johnson. )

JOSEPH RAPHAEL GAMAZ, ætat. 39, born in the city of Carrittano, in the province of Mexico, by trade a taylor, was wounded at Port St. Franciso, on Friday the 24th of January, 1814, at 7 o'clock in the afternoon, while attempting to commit a rape on a female of the place. The wound was inflicted in the left side, under the last floating rib, by a *large clasp knife*, such as is generally carried by the natives in the boot; and which was snatched from the villain at the moment of the assault. It is probable, that the female still kept hold of the knife after it was plunged into the man's side, and considerably enlarged the wound; for, on the knife being withdrawn, the spleen protruded; and in this state it remained till twelve o'clock on Sunday morning following, when it first came under my cognizance.

It was now in a very advanced stage of inflammation, and its organization so much destroyed, that no chance remained of its ever again being restored to vitality, or of its performing its function in the animal economy, whatever that function may be. Its removal, therefore, was determined on; and, as it was only attached to the wound by the vessels of the organ itself, and some cellular substance, a ligature was first applied round them, to prevent hæmorrhage, and then the viscus was removed by the knife.

A very high symptomatic fever ensued, though every part of the most rigorous antiphlogistic treatment was rigidly enforced.

On the 13th of February, the last slough came away, leaving a clean wound, the edges of which were approximated by bandages and adhesive plaister; and on the 20th of March, the man was discharged perfectly cured. From the infliction of the wound till the tenth day, such quantities of blood were voided by urine, as left no doubt of the kidney on that side being wounded by the knife. This was confirmed by an experiment which I made at this time, namely, by plunging a knife into the dead subject, as nearly as possible in the direction of the wound abovementioned; when, on dissection, I found the knife had touched the superior part of the kidney.

I had an opportunity of seeing this man some monhs afterwards, when he appeared, and asserted himself, to be in perfect health.

E. O.

---

" The human spleen, says Dr. Bailie, has even been removed, in a few instances, and the persons have not only recovered, but have enjoyed afterwards, good health." Dr. Bailie does not point out these instances, but I suppose he rests on the authority of Ruysch or Valesnieri.

It is probable, however, that the viscus in question had been in a state of disease previous to its extirpation, in the instances of those authors. The foregoing authentic case, however, cannot but be considered as an interesting fact in physiology, though it by no means establishes the opinion embraced by many, that the human spleen is of no use in the animal economy. What the purposes are which this viscus and the thymus gland answer in the human frame, can only be conjectured; — not demonstrated. Rush's opinion seems by far the most probable in respect to the spleen—namely, that it serves as a receptacle for blood, when, by any accident or disease, the balance of the circulation is broken, and the volume of blood is directed towards the centre of the system from the periphery, as happens from sudden atmospherical transitions, from the cold bath, and particularly during the cold stages of intermittent and ardent fevers. In this point of view, as an organ of preservation, the spleen, though its function be only occasional, is entitled to an importance of rank, much greater than is generally assigned it, while it convinces us, that no part of the human frame is destitute of design or utility.

In respect to the thymus gland, it is the opinion of one of the ablest physicians and physiologists of the present day, that it serves as a diverticulum, when blood is propelled in too great quantity, or with too great momentum, to the head. *Vide, Dr. Parry's Elements of Pathology and Therapeutics, page* 188.

