# Corrigenda

**Published:** 1816-02

**Authors:** 


					CORRIGENDA.
P. 10, 1. 13 for thymus, read thyroid.

				

